# The global prevalence of essential tremor, with emphasis on age and sex: A meta-analysis

**DOI:** 10.7189/jogh.11.04028

**Published:** 2021-04-10

**Authors:** Peige Song, Yan Zhang, Mingming Zha, Qingwen Yang, Xinxin Ye, Qian Yi, Igor Rudan

**Affiliations:** 1 School of Public Health, Zhejiang University School of Medicine, Hangzhou, Zhejiang, China; 2 Women’s Hospital, Zhejiang University School of Medicine, Hangzhou, Zhejiang, China; 3 Faculty of Life Science and Medicine, Kings College London, London, UK; 4 Medical School Southeast University, Nanjing, Jiangsu, China; 5 Centre for Global Health, Usher Institute, University of Edinburgh, Edinburgh, UK

## Abstract

**Background:**

Essential tremor (ET) is one of the most common neurological disorders that impairs quality of life and leads to disability and social handicap. It was estimated that approximate 0.9% of people worldwide were affected by ET. The last decade has seen new investigations on the epidemiology of ET, enabling us to provide an up-to-date estimation of ET prevalence, with emphasis on age and sex.

**Methods:**

We searched PubMed, Medline, Embase and Global Health for studies that reported the prevalence of ET in the general population. Potential sources of heterogeneity were examined by age-adjusted meta-regression. The age- and sex-specific prevalence of ET was constructed with a multilevel mixed-effects meta-regression.

**Results:**

A total of 29 articles were included in our systematic review and meta-analysis. The prevalence of ET increased dramatically with advancing age, where the prevalence estimate in people aged under 20 years was 0.04% (95% confidence interval [CI] = 0.00-0.29) and that in elderly aged 80 years and above was 2.87% (95% CI = 1.07-7.49). ET was consistently more common in males than in females. In 2020, the overall prevalence of ET in the general population was 0.32% (95% CI = 0.12-0.91), and the prevalence was higher in males (0.36%, 95% CI = 0.14-1.03) than in females (0.28%, 95% CI = 0.11-0.79). In 2020, the number of people affected by ET was 24.91million (95% CI = 9.51-70.92), among whom 56% were males.

**Conclusions:**

This study provides an up-to-date estimation of ET prevalence in the general population throughout the whole life span, with emphasis on age and sex. The adoption of an internationally acknowledged diagnostic strategy is prompted in future epidemiological investigations.

**Protocol registration:**

PROSPERO (CRD42020203979)

Essential tremor (ET), primarily characterised by a rhythmic oscillation of agonist and antagonist muscle groups, is one of the most common neurological disorders [1-3]. ET generally involves head, vocal cords and lower limbs, with a variable frequency of 4-12 Hz [4,5]. The traditional conception of ET being a relatively monosymptomatic clinical disorder has been challenged by collective evidence [6,7]. According to the 2018 consensus statement on tremor disorders by the International Parkinson and Movement Disorder Society, ET has been established as a heterogeneous disorder [4,6].

ET affects people of all ages and has a bimodal distribution of age at onset, respectively peaking at the second and sixth decades [2,8]. Although ET is generally regarded as a benign neurological disorder, a wide range of symptoms and comorbidities, including but not limited to intention tremor, gait ataxia, cognitive abnormalities, anxiety and depression, could accumulate as ET progresses, leading to impaired quality of life, disability and social handicap [9,10]. From both the clinical and public health perspectives, reliable estimates of ET prevalence are desirable for guiding adequate treatment, prevention and management.

A systematic review of global prevalence data published up to 2009 was conducted by ED Louis and colleagues in 2010, suggesting that ET affected approximately 0.9% of the world population [3]. Marked heterogeneity between the reported ET prevalence worldwide was noted, largely due to variations in the investigated sample (such as age, sex, ethnicity), case definition and diagnostic approaches, etc. [3,6]. Since then, a growing body of epidemiological investigations on ET has become available during the last decade [11-14]. Until recently, the valuable report by ED Louis and colleagues still serves as by far the best source of quantitative information on the worldwide prevalence of ET, highlighting the need for an updated systematic review and meta-analysis of this topic.

In this study, we conducted an updated systematic review of population-based studies that reported the prevalence of ET in the general population. The primary aim of our study was to determine the global prevalence of ET in the general population based on current evidence. Furthermore, we sought to identify potential associated factors of ET, and to establish the age- and sex-specific prevalence and cases of ET in 2020.

## METHODS

### Search strategy and study selection

This systematic review was performed and reported in accordance with the Preferred Reporting Items for Systematic reviews and Meta-Analysis (PRISMA) [15]. The review protocol was registered online on PROSPERO a prior (CRD42020203979).

By systematically searching four bibliographic databases, namely PubMed, Medline, Embase and Global Health, two authors (MZ and QY) independently identified cross-sectional and longitudinal studies published between January 2000 and December 2019 that reported the prevalence of ET in the general population. Search terms related to ET (“essential tremor”) and prevalence (“prevalence” or “epidemiology”) were combined without language limitations (see Table S1 in the **Online Supplementary Document** for the complete search strategies). In addition, the authors screened the reference lists of relevant systematic reviews to complement the database searches.

Two authors (MZ and QY) independently screened the titles and abstracts of all records, and then reviewed full-texts of all potentially relevant articles to determine eligibility. Studies met inclusion criteria if they were conducted in a sample that was representative of the general population and reported the prevalence of ET (sample size and cases). The study design and diagnostic approaches should have been clearly addressed. Studies were excluded if they were confined to a purposely-selected sample (cognitively impaired patients, people with Parkinson disease, etc.). Multiple publications of the same investigation were compared, and the one with the most comprehensive results was chosen. Discordant results were resolved by discussion between the two authors or by consulting a senior author (PS).

### Data extraction and quality assessment

Three authors (YZ, MZ and QY) independently extracted and cross-checked data from the included articles using a standardised data extraction form. Characteristics of study (authors, publication year, study location, country, World Health Organization [WHO] region, World Bank [WB] region, study period, sampling method, study design) and participants (age range and average age, female proportion, sample size), diagnostic approaches and definitions of ET were collected. Prevalence data, including sample size, ET cases and reported prevalence, were abstracted by age group and sex when available. The WHO regions included African Region (AFR), Region of the Americas (AMR), South-East Asia Region (SEAR), European Region (EUR), Eastern Mediterranean Region (EMR) and Western Pacific Region (WPR), and the WB regions included high-income countries (HIC) and low- and middle-income countries (LMIC). For studies where the investigation years were not available, we assigned them as five years before publication, based on the average time lag between investigation and publication from articles with available information (Table S2 in the **Online Supplementary Document**).

The same three authors evaluated and cross-checked the quality of all included articles independently, according to the Strengthening the Reporting of Observational Studies in Epidemiology (STROBE) guideline [16]. The quality assessment included five domains, namely, sample population, sample size, participation rate, outcome assessment, and analytical methods (Table S3 in the **Online Supplementary Document**). The quality score in each domain ranged from zero to two, and the total score represented the overall bias risk.

### Statistical analysis

In our data extraction stage, one single article might contribute multiple data points (age- or sex-specific prevalence). To best take into account the effect of sample size and fit this hierarchical data structure, we adopted a multilevel mixed-effects meta-regression, with sample size as the weights and the combination of study identification and country identification as the random effect *u_i_* [17-19]. To investigate whether the prevalence of ET varied across different sample characteristics, we first conducted an age-adjusted meta-regression. The a priori selected cluster-level factors included age, sample size, sex (or proportion of female participants), investigation year, study setting (rural or urban), WHO region, WHO region, study design (one-phase, two-phase or three-phase), and participants that were examined (all subjects or the screened positive subjects).

According to the abovementioned models, age, sex and sample size were found to be significantly associated with ET. Given that:

prevalence = *P* = (ET cases) / (number of participants)

Then, the prevalence estimates were first stabilised by the logit transformation,

logit(p) = ln [*p* / (1 – *p*)] = ln(*odds*) = α + β_1_ × *x*_1_ + β_2_ × *x*_2_ + … + *u_i_*

The prevalence of ET was established as a function of age and sex:

logit(p) = α + β_1_ × *Age* + β_2_ × *Female proportion* + *u_i_*

Then,

Prevalence = *P* = e^(^*^α^* ^+^ *^β^*^1 ×^  *^Age^* ^+^ *^β^*^2 ×^  *^Female proportion^* ^+^ *^ui^*^)^ / [1 + e^(^*^α^* ^+^ *^β^*^1 ×^  *^Age^* ^+^ *^β^*^2 ×^  *^Female proportion^* ^+^ *^ui^*^)^]

Finally, the numbers of people living with ET were generated by multiplying the estimated age- and sex-specific prevalence rates by the corresponding world populations in 2020, available from the United Nations Population Division (UNPD) [20].

All analyses were performed using R, version 3.3.0 (R Foundation for Statistical Computing, Vienna, Austria). The significance level was set as *P* < 0.05 in two-sided tests for all analyses.

## RESULTS

### Study selection and characteristics

**Figure 1:**
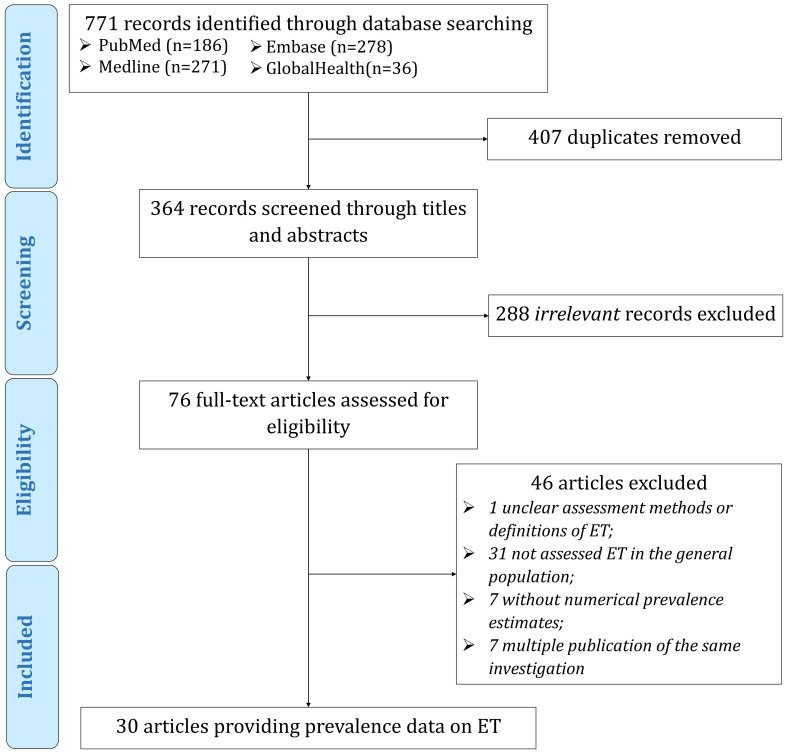
Flowchart of study selection.

**Figure 1** outlines the selection process for the included articles. The initial bibliographic data set searches returned 771 records, 29 of which were included in our meta-analysis. These 29 articles, published between 2001 and 2019, involved a total of 320668 participants and 2250 cases in 13 countries (Brazil, China, Denmark, India, Israel, Italy, South Korea, Nigeria, Singapore, Spain, Tanzania, Turkey, and the United States). Most of the included articles were based on two-phase investigations (screening questionnaire followed by neurological evaluation, n = 21, 72.4%), conducted the diagnostic evaluation in screen-positive subjects (n = 19, 65.6%), and with a quality score of six or above (n = 26, 89.7%). The diagnoses in the included articles were all made by or under the supervision of neurologists or movement disorder specialists. The detailed characteristics and quality appraisal of the identified articles are provided in **Table S4-S5** in the **Online Supplementary Document**.

### Meta-regression

According to the age-adjusted meta-regression models (**Table 1**), advancing age was found to be positively associated with the prevalence of ET, while the prevalence of ET was lower in females than in males (as indicated by both the variables “female proportion” and “sex”). In addition, larger investigations tended to yield lower prevalence estimates, with sample size being negatively associated with the prevalence of ET in our age-adjusted models. However, the prevalence of ET did not significantly differ between rural and urban settings, or among different WB and WHO regions. Moreover, no secular trend in the prevalence of ET was revealed, and study design (one-, two- and three-phase) and diagnostic strategy (in all subjects or only in screened positive subjects) did not seem to have a significant influence on the reported prevalence of ET.

**Table 1:** Age-adjusted meta-regression models of cluster-level factors related to the prevalence of ET (logit form)

Moderator	Number of articles	Number of data points	β	95% CI	*P*-value
**Age**	29	160	0.0534	0.0489 to 0.0578	<0.0001
**Sample size (per 1000)**	29	160	-0.0265	-0.0400 to -0.0129	<0.0001
Investigation year*	29	160	-0.0268	-0.0910 to 0.0374	0.4130
**Female proportion**	29	160	-0.1538	-0.2609 to -0.0466	0.0049
**Sex†**					
Female	21	64	Reference		
Male	21	64	0.1562	0.0490 to 0.2634	0.0043
**Setting**					
Urban	11	64	Reference		
Rural	7	31	-1.1013	-2.3803 to 0.1777	0.0915
**WB region**					
HICs	12	64	Reference		
LMICs	17	96	0.2293	-1.3289 to 1.7875	0.7730
**WHO region**					
WPR	6	35	Reference		
AFR	2	8	-1.1402	-3.9409 to 1.6604	0.4249
AMR	3	14	0.6896	-2.0360 to 3.4151	0.6200
EUR	15	78	0.6125	-1.5419 to 2.7670	0.5774
SEAR	3	25	-0.3832	-3.6722 to 2.9058	0.8194
**Study design**					
One-phase	4	17	Reference		
Two-phase	21	128	-0.0401	-1.1733 to 1.0930	0.9447
Three-phase	3	14	-0.4469	-2.2660 to 1.3723	0.6302
**Who were examined**					
All subjects	8	49	Reference		
Screened positive subjects	19	101	0.2091	-0.8266 to 1.2448	0.6924

ET – essential tremor, WB – World Bank, HIC – high income country, LMIC – low- and middle-income country, WHO – World Health Organization, WPR – Western Pacific Region, AFR – African Region, AMR – Region of the Americas, EUR – European Region, SEAR – South-East Asia Region

*Calendar year.

†The effect of sex was estimated based on studies where sex-specific prevalence was available.

### Age- and sex-specific prevalence and the number of cases of ET in 2020

**Figure 2:**
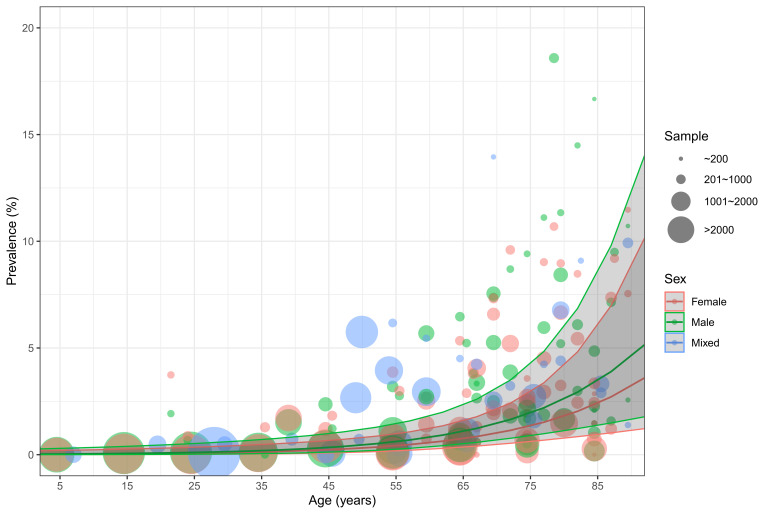
Age- and sex-specific prevalence of essential tremor based on available data points from the included articles, with 95% confidence intervals (CI).

**Figure 3:**
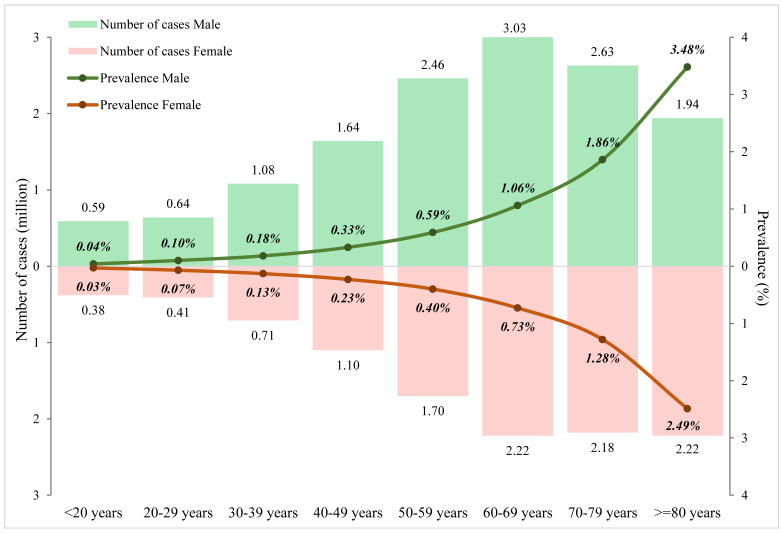
Estimated prevalence and cases of essential tremor in 2020, by age group.

Based on 160 data points from the included articles, the relations of age and sex to the prevalence of ET were established (**Figure 2**). Males consistently had higher prevalence rates than females over the whole life span. In both sexes, the prevalence of ET gradually but slowly increased with advancing age before middle age, starting from less than 0.05% in young people under 20 years of age (0.04%, 95% CI = 0.01-0.35, in males and 0.03%, 95% CI = 0.00-0.23, in females). From 60 years of age onwards, this increasing trend started to become dramatically pronounced, reaching 3.48% (95% CI = 1.32-8.88) and 2.49% (95% CI = 0.92-6.61) in elderly men and women aged 80 years and above respectively. After applying the demographic structure in 2020 (**Table 2** and **Figure 3**), the overall prevalence of ET in the general population was 0.32% (95% CI = 0.12-0.91), and the prevalence was higher in males (0.36%, 95% CI = 0.14-1.03) than in females (0.28%, 95% CI = 0.11-0.79). In 2020, the number of people affected by ET was 24.91million (95% CI = 9.51-70.92), among whom 56% were males.

**Table 2:** Estimated age- and sex-speciﬁc prevalence and cases of ET in 2020

Age group (years)	Prevalence (%, 95% CI)			Cases (million, 95% CI)		
**Male**	**Female**	**Overall**	**Male**	**Female**	**Overall**
<20	0.04	0.03	0.04	0.59	0.38	0.97
	(0.01-0.35)	(0.00-0.23)	(0.00-0.29)	(0.08-4.68)	(0.05-2.90)	(0.13-7.58)
20-29	0.10	0.07	0.09	0.64	0.41	1.04
	(0.02-0.53)	(0.01-0.35)	(0.02-0.44)	(0.12-3.26)	(0.08-2.03)	(0.21-5.29)
30-39	0.18	0.13	0.16	1.08	0.71	1.79
	(0.05-0.71)	(0.03-0.47)	(0.04-0.60)	(0.28-4.18)	(0.19-2.68)	(0.47-6.86)
40-49	0.33	0.23	0.28	1.64	1.10	2.73
	(0.11-1.00)	(0.08-0.66)	(0.09-0.83)	(0.55-4.89)	(0.38-3.20)	(0.92-8.08)
50-59	0.59	0.40	0.50	2.46	1.70	4.16
	(0.24-1.45)	(0.17-0.97)	(0.21-1.21)	(1.00-6.02)	(0.71-4.06)	(1.71-10.09)
60-69	1.06	0.73	0.89	3.03	2.22	5.25
	(0.48-2.32)	(0.33-1.57)	(0.40-1.94)	(1.37-6.65)	(1.02-4.81)	(2.39-11.46)
70-79	1.86	1.28	1.54	2.63	2.18	4.81
	(0.82-4.13)	(0.57-2.85)	(0.68-3.43)	(1.17-5.86)	(0.97-4.86)	(2.14-10.72)
≥80	3.48	2.49	2.87	1.94	2.22	4.16
	(1.32-8.88)	(0.92-6.61)	(1.07-7.49)	(0.73-4.96)	(0.82-5.90)	(1.55-10.85)
Overall	0.36	0.28	0.32	14.01	10.90	24.91
	(0.14-1.03)	(0.11-0.79)	(0.12-0.91)	(5.31-40.49)	(4.20-30.43)	(9.51-70.92)

ET – essential tremor, CI – confidence interval

## DISCUSSION

This systematic review and meta-analysis of 29 articles published from 2001 to 2019 provided a comprehensive and up-to-date assessment of the prevalence of ET worldwide. Based on a substantial number of data points from included articles, we revealed that the prevalence of ET increased with advancing age, predominantly in elderly aged 60 years and above. Moreover, ET was found to be consistently more common in males than in females across the whole life span. In 2020, the global prevalence of ET was 0.32% in the general population, ranging from 0.04% in people under 20 years to 2.87% in those aged 80 years and above. The total number of people suffering from ET worldwide was 24.91 million in 2020.

The previous systematic review and meta-analysis by ED Louis and colleagues reported a pooled ET prevalence of 0.9%, which was much higher than our prevalence estimate of 0.32%. As revealed in our meta-regression and widely in previous investigations, the prevalence of ET increased exponentially with advancing age [3,21,22]. Given that more than half of the included studies in the meta-analysis by ED Louis and colleagues were conducted in middle-aged (≥40 years) or elderly (≥60 years), it is therefore not surprising to observe a relatively higher than expected ET prevalence by their estimation. The great variety of reported ET prevalence according to age group leads to the necessity of an age-specific estimation in data synthesis, which led to the initiation and then became the most obvious merit of our study. Apart from age, the sex difference in ET prevalence is a topic of both clinical and public health interests. Several previous investigations have revealed no sex differences in ET prevalence, while some others demonstrated that the prevalence was higher in males than in females [3,5]. ET disproportionally affect different regions of the body between sexes, with head and voice tremor being more frequent among females and postural hand tremor being more common and more severe among males [23]. After controlling the effect of age, a significantly higher prevalence of ET in males was revealed in our study, which is consistent with findings in previous primary investigations, especially in the paediatric population [3,24]. This phenomenon might be resulted by the impact of sex chromosomes and sex hormones [23,25]. Moreover, the possible pathological associations between ET and Parkinson disease and the fact that Parkinson disease is more frequent in males might also accumulate more ET cases in males than in females [26].

To the best of our knowledge, this study provides the first age- and sex-specific prevalence estimates of ET throughout the whole life span, based on up-to-date epidemiological evidence. Benefiting from our comprehensive search strategy, we included 29 articles published after 2000. The sufficient data points from the included articles guaranteed our availability to construct the age- and sex-specific prevalence estimates of ET, and ensured the power of our statistical analyses. Another strength of our study was the representativeness of our estimates of the whole population. After controlling for the effect of age, other cluster-level factors, including setting (urban vs rural), WB region, WHO regions, study design (one-, two- and three-phase) and even diagnostic strategy (in all subjects or only in screened positive subjects) did not seem to influence the prevalence of ET significantly, reinforcing the predominant role of age as the greatest driver of ET [21].

Several potential limitations should be considered when interpreting the results of our study. First, the majority of our included articles were based on two- or three-phase investigations and only conducted neurological examinations in screen-positive subjects. Although our meta-regression did not indicate study design and diagnostic strategy as significant associated factors, we could not fully rule out the heterogeneity introduced by variations in studies with different designs. Despite the fact that the diagnoses of ET in the included articles were all made by or confirmed by neurologists or movement disorder specialists, an underestimation could still be possible given the questionnaire-based screening during the first phase of case ascertainment might have omitted a number of positive cases. In line with studies on other neurological disorders, such as Tourette syndrome and epilepsy, epidemiological investigations involving larger sample sizes tend to report relatively lower prevalence [27,28]. Second, to lower the effect of outliers with small sample sizes, we weighted contributing data points by sample size. Since larger investigations generally adopted two- or three-phase study designs without a throughout assessment of all participants, overweighting larger studies might bring underestimated prevalence. Third, we only evaluated the effects of a limited number of variables that were commonly available in the included articles, many unexamined factors, such as ethnicity, ET subtype, age-of-onset, might have contributed to the heterogeneity among included studies. A better understanding of the epidemiological distribution of ET across the world should involve more covariates, when relevant evidence becomes available in the foreseeable future.

The findings in our study have both clinical and public health implications. The lack of agreed and shared tools and definitions in studies on ET has been highlighted, emphasising the need for adopting an internationally acknowledged diagnostic approach in future epidemiological investigations in ET [4]. Moreover, new epidemiological studies should try to address different subtypes of ET, if possible. Considering that older adults, and men more than women, are at a substantially higher risk of ET compared to their younger counterparts, more attention to clinical diagnostics and treatment should be given to this population once the symptoms of ET appear.

## CONCLUSIONS

In conclusion, our study provides a comprehensive estimation of ET prevalence to date, with emphasis on age and sex. ET became more common with advancing age, especially in elderly. Males were more likely to have ET than females. The adoption of an internationally acknowledged diagnostic strategy is prompted in future epidemiological investigations. Given the prominent role of ageing in the development of ET and the global ageing context, more people affected by ET are expected in the foreseeable future.

## Additional material

Online Supplementary Document
