# Supplementary material for: The global prevalence of essential tremor, with emphasis on age and sex: A meta-analysis
Source: J Glob Health. 2021 Apr 10;11:04028. doi: 10.7189/jogh.11.04028 (PMC8035980; doi:10.7189/jogh.11.04028)
Supplement: Online Supplementary Document [file jogh-11-04028-s001.pdf]

Supplementary Appendix

Song et al. The global prevalence of essential tremor, with emphasis on age and sex: a meta-analysis

Table S1. Search strategy to identify studies reporting the prevalence of essential tremor in the general population

Table S2. The time lag between investigation and publication in the included articles reporting the prevalence of essential tremor in the general population (n=29)

Table S3. Quality assessment scale for rating the risk of bias

Table S4. Detailed characteristics of the included articles (n=29)

Table S5. Quality scores for assessing the risk of bias in the included articles (n=29)

This supplementary material has been provided by the authors to give readers additional information about their work.

Table S1. Search strategy to identify studies reporting the prevalence of essential tremor in the general population

Database	Access date	Search terms
**PubMed**	21st Dec, 2019	(Essential Tremor [Title/Abstract]) AND (prevalence[Title/Abstract] OR epidemiology[Title/Abstract]) AND ("2000/01/01"[Date - Publication] : "3000"[Date - Publication])<br>Filter: Humans
**MEDLINE (1950-)**	21st Dec, 2019	1 exp Essential Tremor/<br>2 Essential Tremor.ab,ti.<br>3 (prevalen* or epidemiolog*).ab,ti.<br>4 1 or 2<br>5 3 and 4<br>6 limit 5 to (humans and yr="2000 -Current" and medline)
**EMBASE (1980-)**	21st Dec, 2019	1 exp essential tremor/<br>2 Essential Tremor.ab,ti.<br>3 (prevalen* or epidemiolog*).ab,ti.<br>4 1 or 2<br>5 3 and 4<br>6 limit 5 to (human and embase and yr="2000 -Current" and (article or article in press or short survey) and journal)
**Global Health (1973-)**	21st Dec, 2019	1 Essential Tremor.ab,ti.<br>2 (prevalen* or epidemiolog*).ab,ti.<br>3 1 and 2<br>4 limit 3 to yr="2000 -Current"

Table S2. The time lag between investigation and publication in the included articles reporting the prevalence of essential tremor in the general population (n=29)

Study ID	Author(s)	Year of publication	Year of investigation	Time-lag (year)
1[1]	Bergareche A, et al.	2001	NS	
2[2]	Benito-León J, et al.	2003	1994	9
3[3]	Dogu O, et al.	2003	2002	1
4[4]	Louis ED, et al.	2003	1998.5	4.5
5[5]	Dogu O, et al.	2004	NS	
6[6]	Tan LCS, et al.	2005	2002	3
7[7]	Inzelberg R, et al.	2006	2003	3
8[8]	Sun H, et al.	2006	2000	6
9[9]	Mancini ML, et al.	2007	NS	
10[10]	Das SK, et al.	2008	2003	5
11[11]	Dotchin CL, et al.	2008	2005	3
12[12]	Das SK, et al.	2009	2003	6
13[13]	Erer S, et al.	2009	2004	5
14[14]	Glik A, et al.	2009	NS	
15[15]	Kusbeci OY, et al.	2009	NS	
16[16]	Louis ED, et al.	2009	2000	9
17[17]	Sur H, et al.	2009	2005	4
18[18]	Liu Y, et al.	2011	2008	3
19[19]	Aharon-Peretz J, et al.	2012	NS	
20[20]	Okubadejo NU, et al.	2012	2008	4
21[21]	Barbosa MT, et al.	2013	2001.5	11.5
22[22]	Ozel L, et al.	2013	NS	
23[23]	Seijo-Martinez M, et al.	2013	2004	9
24[24]	Oh ES, et al.	2014	2006	8
25[25]	Yani Y, et al.	2015	2015	0
26[26]	Wu Y, et al.	2016	2012	4
27[27]	Mansukhani KA, et al.	2018	NS	
28[28]	Eliasen EH, et al.	2019	2017	2
29[29]	Guler S, et al.	2019	2013	6

Note: NS=not specifirf; The average time-lag between investigation and publication was 5.05 based on 21 articles with available data.

Table S3. Quality assessment scale for rating the risk of bias

Bias type	Low risk (score=2)	Moderate risk (score=1)	High risk (score=0)
Selection (sample population)	1) Sample from the general population, not a select group;<br>2) Consecutive unselected population;<br>3) Rationale for case and control selection explained.	1) Sample selected from large population but selection criteria not defined;<br>2) Sample selection ambiguous but may be representative;<br>3) Rationale for cases and controls not explained;<br>4) Eligibility criteria not explained;<br>5) Analysis to adjust for sampling strategy bias.	1) Highly select population making it difficult to generalise finding;<br>2) Sample selection ambiguous and sample unlikely to be representative.
Selection (sample size)	1) Sample size calculation performed and adequate.	1) Sample size calculation performed and reasons for not meeting sample size given;<br>2) Sample size calculation not performed but all eligible persons studied.	1) Sample size estimation unclear or only sub-sample studied.
Selection (participation rate)	1) High response rate (>85%).	1) Moderate response rate (70-85%).	1) Low response rate (<70%);<br>2) Response rate not reported.
Performance bias (outcome assessment)	1) Diagnosis using consistent criteria and direct examination.	1) Assessment from administrative database or register;<br>2) Assessment from hospital record or interviewer.	1) Assessment from non-validated data or generic estimate from the overall population.
Performance bias (analytical methods to control for bias)	1) Analysis appropriate for the type of sample (subgroup analysis/regression etc.).	1) Analysis does not account for common adjustment.	1) Data confusing.

Table S4. Detailed characteristics of the included articles (n=29)

ID	Author (s)	Year Published	Country	WHO	WB	Study setting	Urban or rural	Investigation Date	Sampling Strategy	Study design	Screened by whom	Who were examined	Diagnosed by whom	Definition of ET	Sample size	Cases	Age range	Female proportion
1[1]	Bergareche A, et al.	2001	Spain	EUR	HIC	Irun and Hondarribia, in the north of Guipuzcoa	mixed	NS	Aleatory stratified sampling	Two-phase	Door-to-door by trained interviewers	screened positive	neurologists	We defined ET as a postural or kinetic tremor of the head or limbs without a recognizable cause. The tremor had to be gradual in onset, present for at least 1 year, or accompanied by family history of the same disorder if present for less than 1 year (at least one first-degree relative affected)	1540	48	65+	0.60
2[2]	Benito-León J, et al.	2003	Spain	EUR	HIC	Margaritas in Getafe; Lista in Salamanca district; and Arévalo of Arévalo county	both	1994	Proportionate stratified random sampling	Two-phase	medically unsophisticated interviewers in subjects' homes or at nearby clinics	screened positive	Eight trained neurologists	Subjects were diagnosed as having ET if they had an action tremor of the head, limbs, or voice without any recognizable cause. The tremor had to be of gradual onset and either present for at least 1 year, or accompanied by a family history of the same disorder (at least one first-degree relative affected). Subjects with tremor related to alcohol withdrawal, hyperthyroidism, anxiety, Parkinson's disease, antidopaminergic drug in- take, lithium therapy, or other known causes of tremor,were excluded.	5278	256	65+	0.58
3[3]	Dogu O, et al.	2003	Turkey	EUR	UMIC	Mersin	both	2002/07-2002/12	Random sampling	Two-phase	Door-to-door by neurologists	all subjects	neurologists	the presence of moderate or greater amplitude kinetic tremor during three or more tests or a head tremor were defined as ET.	2253	89	40+	0.50
4[4]	Louis ED, et al.	2003	United States	AMR	HIC	Pittsburgh, Forsyth County,	urban	1998-1999	Clustered sampling	One phase+physician	Self-report	physician diagnosis	previous physician-diagnosis	NS	3494	54	70-103	0.62

ID	Author (s)	Year Published	Country	WHO	WB	Study setting	Urban or rural	Investigation Date	Sampling Strategy	Study design	Screened by whom	Who were examined	Diagnosed by whom	Definition of ET	Sample size	Cases	Age range	Female proportion
						Sacramento County, and Washington County				diagnosis of ET								
5[5]	Dogu O, et al.	2004	Turkey	EUR	UMIC	Mersin	urban	NS	Random sampling	Two-phase	Door-to-door by neurologists	all subjects	neurologists	moderate-amplitude kinetic tremor during a minimum of 3 tests or a head tremor without signs of dystonia or Parkinson disease	2253	89	40+	NS
6[6]	Tan LCS, et al.	2005	Singapore	WPR	HIC	Ang Mo Kio, Bishan, Toa Payoh, Serangoon, and Yishun	mixed	2001-2003	Clustered sampling	Three-phase	Trained interviewers	screened positive	a movement disorders specialist (LT) or a trained fellow (VR)	the presence of bilateral action (postural or kinetic) tremor of the hands and forearms in the absence of other neurologic signs (with the exception of the cogwheel phenomena), or the presence of isolated head tremor with no signs of dystonia	14906	40	50+	0.54
7[7]	Inzelberg R, et al.	2006	Israel	EUR	HIC	Arabic villages fo Wadi Ara in Northern Israel	rural	2003	Clustered sampling	Two-phase	a nurse	all subjects	a neurologist	Diagnosis of definite ET required moderate oscillatory postural tremor usually present during the examination, moderate and clearly oscillatory kinetic tremor in at least one arm during four of five actions and tremor that by history interfered with ≥1 activity of daily living. The diagnosis of probable ET required a moderate, clearly oscillatory kinetic tremor usually present during examination and during four of the five actions. Possible ET required a moderate clearly oscillatory kinetic tremor during the action.	428	8	65+	0.46

ID	Author (s)	Year Published	Country	WHO	WB	Study setting	Urban or rural	Investigation Date	Sampling Strategy	Study design	Screened by whom	Who were examined	Diagnosed by whom	Definition of ET	Sample size	Cases	Age range	Female proportion
8[8]	Sun H, et al.	2006	China	WPR	UMIC	Beijing	both	2000	Clustered sampling	Two-phase	Trained interviewers	screened positive	neurologists	definition in the NIH ET diagnosis criteria	2835	135	55+	0.51
9[9]	Mancini ML, et al.	2007	Italy	EUR	HIC	Lake Trasimeno, in the Umbria region of central Italy	mixed	NS	Clustered sampling	Two-phase	general practitioners	all subjects	physicians, under supervision of a neurologist and being checked by a movement disorder expert	For inclusion in the study, both major criteria were considered mandatory, whereas one or more minor criteria were used as adjuvant criteria for ET diagnosis.	13604	108	27-110	0.48
10[10]	Das SK, et al.	2008	India	SEAR	LMIC	Kolkata	urban	2003/03-2004/02	Random sampling	Two-phase	a doctor who was a neurologist, a neuropsychologist and four field workers	screened positive	senior neurologists and psychiatrist	Essential tremor based on the consensus statement of the movement disorders society on tremor (1998). Bilateral action tremor of hands and forearms or isolated head tremor with no abnormal posture for at least three years with or without positive family history without any neurological sign except cogwheel phenomenon.	5430	75	60+	0.49
11[11]	Dotchin CL, et al.	2008	Tanzania	AFR	LMIC	Hai District	rural	2005/08	Clustered sampling	Two-phase	research doctor	screened positive	research doctor	ET was diagnosed in patients with a postural or kinetic tremor of the upper limbs, and/or an isolated head tremor, with no other focal neurology	161071	65	40-96	NS
12[12]	Das SK, et al.	2009	India	SEAR	LMIC	Kolkata	urban	2003/02-2004/04	Random sampling	Two-phase	field investigators	screened positive	neurologist	The operational definition of ET included bilateral, largely symmetric, postural or kinetic tremor involving hands and forearms that is visible and persistent; additional or isolated	52377	184	all ages	0.47

ID	Author (s)	Year Published	Country	WHO	WB	Study setting	Urban or rural	Investigation Date	Sampling Strategy	Study design	Screened by whom	Who were examined	Diagnosed by whom	Definition of ET	Sample size	Cases	Age range	Female proportion
														tremor of the head was considered in the absence of abnormal posturing.				
13 [13]	Erer S, et al.	2009	Turkey	EUR	UMIC	Orhangazi district	both	2004/06-2005/05	Clustered sampling	Three-phase	3 family medicine and 3 neurology residents	screened positive	movement disorder specialists	Essential tremor (ET), the most common adult tremor, is primarily an autosomal dominant disease characterized by postural and kinetic tremor of body parts without other neurological signs.	1124	21	40-95	0.51
14 [14]	Glik A, et al.	2009	Israel	EUR	HIC	Wadi Ara Arabic villages in Norther Israel	rural	NS	Clustered sampling	One-phase	NS	all subjects	neurologists	Diagnosis of definite ET required moderate oscillatory postural tremor usually present during examination, a moderate and clearly oscillatory kinetic tremor in at least one arm during 4/5 actions and tremor that by history interfered with ≥1 ADL.	918	7	65+	0.51
15 [15]	Kusbeci OY, et al.	2009	Turkey	EUR	UMIC	Afyon Kocatepe University School of Medicine	urban	NS	Clustered sampling	Two-phase	five medical school students who were trained for ET	screened positive	a neurology specialist	Subjects who had action tremor of the head, limbs or voice without recognizable causes were diagnosed as having ET.	221	6	17-26	0.53
16 [16]	Louis ED, et al.	2009	United States	AMR	HIC	Northern Manhattan	urban	1999-2001	Aleatory stratified sampling	Two-phase	A trained research assistant and a general physician	all subjects	senior neurologist	A final diagnosis of ET was assigned when the senior neurologist confirmed a total tremor score of 5.5 or higher or rated the handwritten sentence ≥ 2.	2776	108	66-102	0.66
17 [17]	Sur H, et al.	2009	Turkey	EUR	UMIC	Şile	rural	2005/05-2005/07	Random sampling	Two-phase	a specialist and a resident	all subjects	two neurologists	Based on the interview and examination, each neurologist independently diagnosed the subjects as ET or normal. A final diagnosis of	2227	69	18-104	0.58

ID	Author (s)	Year Published	Country	WHO	WB	Study setting	Urban or rural	Investigation Date	Sampling Strategy	Study design	Screened by whom	Who were examined	Diagnosed by whom	Definition of ET	Sample size	Cases	Age range	Female proportion
														ET was assigned upon agreement of both neurologists.				
18 [18]	Liu Y, et al.	2011	China	WPR	UMIC	Kashkar	both	2008/04-2009/07	Clustered sampling	Two-phase	neurologist	screened positive	a neurologist and a senior physician	NS	2834	184	55-92	0.51
19 [19]	Aharon-Peretz J, et al.	2012	Israel	EUR	HIC	Druze villages of the Galilee	rural	NS	Clustered sampling	Two-phase	medical nurse by profession and trained by a senior clinical investigator or specializing in movement disorders	screened positive	a senior neurologist	Subjects were diagnosed as having ET if they reported tremor of the head, limbs, or voice without any other recognizable cause. The tremor had to be of gradual onset (i.e., slow and progressive) and had to have been present for at least 1 year or be accompanied by a family history of the same disorder.	3980	27	60+	0.52
20 [20]	Okubadejo NU, et al.	2012	Nigeria	AFR	LMIC	Surulere Local Government Area (LGA), Lagos State, Nigeria	urban	2008/03-2008/09	Random sampling	Two-phase	NS	screened positive	neurologist	ET was diagnosed in the presence of both major criteria (bilateral action tremor of the hands and forearms or isolated head tremor without dystonia) in the absence of any other neurological signs.	3000	36	all ages	0.44
21 [21]	Barbosa MT, et al.	2013	Brazil	AMR	UM	Bambuí	urban	2001-2002	Clustered sampling	Two-phase	trained interviewers	random sample of individua	movement disorder-trained	We defined and classified tremor, including enhanced physiological tremor, according to the consensus	1186	122	64-98	0.62

ID	Author (s)	Year Published	Country	WHO	WB	Study setting	Urban or rural	Investigation Date	Sampling Strategy	Study design	Screened by whom	Who were examined	Diagnosed by whom	Definition of ET	Sample size	Cases	Age range	Female proportion
					IC							ls who screened negative in phase 1 and individuals who screened positive in phase 2	neurologists or geriatricians	statement of the Movement Disorder Society (MDS) for a clinical classification of tremors.				
22 [22]	Ozel L, et al.	2013	Turkey	EUR	UMIC	Erzurum city center	urban	NS	Random sampling	Three-phase	trained resident physicians	screened positive	a faculty member specializing in movement disorders	NS	3960	64	18-60	0.50
23 [23]	Seijo-Martinez M, et al.	2013	Spain	EUR	HIC	Arosa Island	mixed	2004	Clustered sampling	One phase	NS	screened positive	neurologists	NS	753	65	65+	0.58
24 [24]	Oh ES, et al.	2014	Korea	WPR	HIC	Seongnam City	urban	2005-2007	Clustered sampling	Two-phase	physicians	all subjects	a movement disorder specialist	NS	714	26	65-99	0.58
25 [25]	Yani Y, et al.	2015	China	WPR	UMIC	Chongwen, Yueguang, Qingnian and Hongqiao in Urumqi	urban	2015	Random sampling	One phase	NS	screened positive	neurologists	NS	5463	198	45+	0.48

ID	Author (s)	Year Published	Country	WHO	WB	Study setting	Urban or rural	Investigation Date	Sampling Strategy	Study design	Screened by whom	Who were examined	Diagnosed by whom	Definition of ET	Sample size	Cases	Age range	Female proportion
26 [26]	Wu Y, et al.	2016	China	WPR	UMIC	Malu town in northwestern Shanghai	urban	2011/04-2013/10	Clustered sampling	Two-phase	trained physicians	screened positive	movement disorder specialists	NS	19614	60	50+	0.52
27 [27]	Mansukhani KA, et al.	2018	India	SEAR	LMIC	the villages of Moti Vahiyal, Arnai, and Chavshala in Kaparada taluka in the Valsad district	rural	NS	Clustered sampling	Two-phase	trained volunteers	screened positive	neurologists	NS	8217	6	all ages	0.49
28 [28]	Eliasen EH, et al.	2019	Denmark	EUR	HIC	Faroe Islands	mixed	2016/08-2017/12	Random sampling	Two-phase	NS	screened positive	a senior movement disorder neurologist with particular specialization in tremor	moderate or greater amplitude kinetic tremor during 3 or more activities or a head tremor in the absence of PD or another known cause [e.g., medication-induced tremor, tremor from hyperthyroidism]	1328	27	40+	0.53
29 [29]	Guler S, et al.	2019	Turkey	EUR	UMIC	Edirne and its districts	rural	2013	Random sampling	NS	Clinical Evaluation Enrollees	screened positive	neurologist specializing in movement disorders	participants were diagnosed with ET if they had an action tremor of the hand, head, limbs, foot or voice without any other recognizable cause. Second, the tremor had to be of gradual onset (i.e. slow and	3008	173	30+	0.50

ID	Author (s)	Year Publi she d	Cou ntry	W H O	W B	Study setting	Urban or rural	Inves tigati on Date	Sampling Strategy	Study design	Screened by whom	Who were examine d	Diagnosed by whom	Definition of ET	Sa mp le siz e	C a s e s	Ag e ra ng e	Femal e propo rtion
														progressive) and (1) present for at least 1 year or (2) accompanied by a family history of the same disorder (at least one reportedly affected the first-degree relative).				

Note: NS=not specified; WHO=World Health Organization; WB=World Bank; HIC=high-income countries; LMIC= low- and middle-income countries; UMIC= upper middle-income countries; For articles that didn't specify the setting of investigations (urban or rural), their settings were recorded as "mixed".

Table S5. Quality scores for assessing the risk of bias in the included articles (n=29)

ID	Author(s)	Year Published	Quality score					
			Sample population	Sample size	Participation	Outcome assessment	Analytical methods	Total scores
1[1]	Bergareche A, et al.	2001	2	1	2	2	2	9
2[2]	Benito-León J, et al.	2003	2	2	2	2	1	9
3[3]	Dogu O, et al.	2003	2	2	2	2	1	9
4[4]	Louis ED, et al.	2003	2	1	0	0	0	3
5[5]	Dogu O, et al.	2004	2	1	1	0	0	4
6[6]	Tan LCS, et al.	2005	2	2	2	2	2	10
7[7]	Inzelberg R, et al.	2006	2	2	2	2	1	9
8[8]	Sun H, et al.	2006	2	2	0	2	1	7
9[9]	Mancini ML, et al.	2007	2	2	2	0	2	8
10[10]	Das SK, et al.	2008	2	2	2	1	1	8
11[11]	Dotchin CL, et al.	2008	2	2	2	0	1	7
12[12]	Das SK, et al.	2009	2	2	2	1	1	8
13[13]	Erer S, et al.	2009	2	2	2	2	2	10
14[14]	Glik A, et al.	2009	1	1	2	2	1	7
15[15]	Kusbeci OY, et al.	2009	2	2	2	2	2	10
16[16]	Louis ED, et al.	2009	2	2	2	2	1	9
17[17]	Sur H, et al.	2009	2	2	1	2	1	8
18[18]	Liu Y, et al.	2011	2	2	2	1	1	8
19[19]	Aharon-Peretz J, et al.	2012	1	1	2	2	1	7
20[20]	Okubadejo NU, et al.	2012	1	2	2	1	1	7
21[21]	Barbosa MT, et al.	2013	2	2	2	1	1	8
22[22]	Ozel L, et al.	2013	2	2	2	1	1	8
23[23]	Seijo-Martinez M, et al.	2013	2	2	2	1	2	9
24[24]	Oh ES, et al.	2014	2	1	0	1	1	5
25[25]	Yani Y, et al.	2015	2	2	2	1	2	9
26[26]	Wu Y, et al.	2016	2	2	2	2	2	10
27[27]	Mansukhani KA, et al.	2018	2	2	2	0	0	6
28[28]	Eliasen EH, et al.	2019	2	2	2	1	2	9
29[29]	Guler S, et al.	2019	2	2	2	0	0	6

References

1 Bergareche A, De La Puente E, López De Munain A, Sarasqueta C, De Arce A, Poza JJ, et al. Prevalence of essential tremor: a door-to-door survey in bidasoa, spain. Neuroepidemiology. 2001;20:125-8.

2 Benito-León J, Bermejo-Pareja F, Morales J-M, Vega S, Molina J-A. Prevalence of essential tremor in three elderly populations of central Spain. Mov Disord. 2003;18:389-94.

3 Dogu O, Sevim S, Camdeviren H, Sasmaz T, Bugdayci R, Aral M, et al. Prevalence of essential tremor: door-to-door neurologic exams in Mersin Province, Turkey. Neurology. 2003;61:1804-6.

4 Louis ED, Fried LP, Fitzpatrick AL, Jr. Longstreth WT, Newman AB. Regional and racial differences in the prevalence of physician-diagnosed essential tremor in the United States. Mov Disord. 2003;18:1035-40.

5 Dogu O, Sevim S, Louis ED, Kaleagasi H, Aral M. Reduced body mass index in patients with essential tremor: a population-based study in the province of Mersin, Turkey. Arch Neurol . 2004;61:386-9.

6 Tan LCS, Venketasubramanian N, Ramasamy V, Gao W, Saw S-M. Prevalence of essential tremor in Singapore: a study on three races in an Asian country. Parkinsonism Relat Disord. 2005;11:233-9.

7 Inzelberg R, Mazarib A, Masarwa M, Abuful A, Strugatsky R, Friedland RF. Essential tremor prevalence is low in Arabic villages in Israel: door-to-door neurological examinations. J Neurol. 2006;253:1557-60.

8 Sun H, Chen B, Sun F, Zhang XQ, Tang Z, Liu HJ, et al. Prevalence of essential tremor in community of elderly people in Beijing. Chin J Neurol. 2006;39:189-92.

9 Mancini ML, Stracci F, Tambasco N, Sarchielli P, Rossi A, Calabresi P. Prevalence of essential tremor in the territory of Lake Trasimeno, Italy: results of a population-based study. Mov Disord. 2007;22:540-5.

10 Das SK, Biswas A, Roy J, Bose P, Roy T, Banerjee TK, et al. Prevalence of major neurological disorders among geriatric population in the metropolitan city of Kolkata. J Assoc Physicians India. 2008;56:175-81.

11 Dotchin CL, Walker RW. The prevalence of essential tremor in rural northern Tanzania. J Neurol Neurosurg Psychiatry. 2008;79:1107-9.

12 Das SK, Banerjee TK, Roy T, Raut DK, Chaudhuri A, Hazra A. Prevalence of essential tremor in the city of Kolkata, India: a house-to-house survey. Eur J Neurol. 2009;16:801-7.

13 Erer S, Zarifoglu M, Karli N, Ozcakir A, Cavdar C, Ocakoglu G. Clinical characteristics of essential and physiological tremor in Orhangazi district of Bursa, Turkey: A population based study. J Neurol Sci. 2009;26:120-30.

14 Glik A, Masarwa M, Abuful A, Deeb A, Strugatsky R, Farrer LA, et al. Essential tremor might be less frequent than Parkinson's disease in North Israel Arab villages. Mov Disord. 2009;24:119-22.

15 Kusbeci OY, Ayhan S, Bayram FB, Esenboga T, Yapa AB, Coker B, et al. The prevalence of essential tremor in Turkish Medical Students. J Neurol Sci. 2009;26:56-9.

16 Louis ED, Thawani SP, Andrews HF. Prevalence of essential tremor in a multiethnic, community-based study in northern Manhattan, New York, N.Y. Neuroepidemiology. 2009;32:208-14.

17 Sur H, Ilhan S, Erdoğan H, Oztürk E, Taşdemir M, Börü UT. Prevalence of essential tremor: a door-to-door survey in Sile, Istanbul, Turkey. Parkinsonism Relat Disord. 2009;15:101-4.

18 Liu Y, Zhang X-y, Tang Y-z, He Y, Han X-H, Guo M, et al. Investigation on prevalence rate of essential tremor in population aged 55 years old and above in Kashkar, between 2008 and 2009. Chin Med J. 2011;91:1067-9.

19 Aharon-Peretz J, Badarny S, Ibrahim R, Gershoni-Baruch R, Hassoun G. Essential tremor prevalence is low in the druze population in northern Israel. Tremor Other Hyperkinet Mov. 2012;1.
20 Okubadejo NU, Bankole IA, Ojo OO, Ojini FI, Danesi MA. Prevalence of essential tremor in urban Lagos, Nigeria: a door-to-door community-based study. BMC Neurol. 2012;12:110-.
21 Barbosa MT, Caramelli P, Cunningham MCQ, Maia DP, Lima-Costa MFF, Cardoso F. Prevalence and clinical classification of tremor in elderly--a community-based survey in Brazil. Mov Disord. 2013;28:640-6.
22 Ozel L, Demir R, Ozdemir G, Ozyıldırım E, Avşar U, Ulvi H, et al. Investigation of the prevalence of essential tremor in individuals aged 18-60 in Erzurum. Acta Neurol Belg. 2013;113:127-31.
23 Seijo-Martinez M, Del Rio MC, Rodriguez Alvarez JR, Suarez Prado R, Torres Salgado E, Paz Esquete J, et al. Prevalence of essential tremor on Arosa Island, Spain: A community-based, door-to-door survey. Tremor Other Hyperkinet Mov. 2013;1.
24 Oh ES, Kim J-M, Kim YE, Yun JY, Kim JS, Kim SE, et al. The prevalence of essential tremor in elderly Koreans. J Korean Med Sci. 2014;29:1694-8.
25 Yani Y, Yuling W, Xinling Y. Related factors and prevalence for the essential tremor disease of uygur residents in Hetian, Xinjiang uygur autonomous region. Chin Med J. 2015;95:69-72.
26 Wu Y, Wang X, Wang C, Sun Q, Song N, Zhou Y, et al. Prevalence and clinical features of non-motor symptoms of essential tremor in Shanghai rural area. Parkinsonism Relat Disord. 2016;22:15-20.
27 Mansukhani KA, Barretto MA, Donde SA, Wandrekar J, Nigudkar A, Nair R. Epidemiological survey of neurological diseases in a tribal population cluster in Gujarat. Ann Indian Acad Neurol. 2018;21:294-9.
28 Eliasen EH, Ferrer M, Gaini S, Louis ED, Petersen MS. Prevalence of Essential Tremor in the Faroe Islands: A Population-Based Study. Neuroepidemiology. 2019:227-35.
29 Guler S, Caylan A, Turan F, Dagdeviren N. The prevalence of essential tremor in Edirne and its counties accompanied comorbid conditions. Neurol Res. 2019;41:847-56.
